# Prediction of future labour market outcome in a cohort of long-term sick- listed Danes

**DOI:** 10.1186/1471-2458-14-494

**Published:** 2014-05-23

**Authors:** Jacob Pedersen, Thomas Alexander Gerds, Jakob Bue Bjorner, Karl Bang Christensen

**Affiliations:** 1 National Research Centre for the Working Environment (NRCWE), Lersø Parkallé 105, DK-2100, Copenhagen Ø, Denmark; 2 Department of Public Health, University of Copenhagen, Copenhagen, Denmark; 3 QualityMetric, Lincoln, RI, USA

**Keywords:** Labour market, Long-term sick-listed, Risk profiling, Logistic regression, Discrete event simulation, Register data, Registry

## Abstract

**Background:**

Targeted interventions for the long-term sick-listed may prevent permanent exclusion from the labour force. We aimed to develop a prediction method for identifying high risk groups for continued or recurrent long-term sickness absence, unemployment, or disability among persons on long-term sick leave.

**Methods:**

We obtained individual characteristics and follow-up data from the Danish Register of Sickness Absence Compensation Benefits and Social Transfer Payments (RSS) during 2004 to 2010 for 189,279 Danes who experienced a period of long-term sickness absence (4+ weeks). In a learning data set, statistical prediction methods were built using logistic regression and a discrete event simulation approach for a one year prediction horizon. Personalized risk profiles were obtained for five outcomes: employment, unemployment, recurrent sickness absence, continuous long-term sickness absence, and early retirement from the labour market. Predictor variables included gender, age, socio-economic position, job type, chronic disease status, history of sickness absence, and prior history of unemployment. Separate models were built for times of economic growth (2005–2007) and times of recession (2008–2010). The accuracy of the prediction models was assessed with analyses of Receiver Operating Characteristic (ROC) curves and the Brier score in an independent validation data set.

**Results:**

In comparison with a null model which ignored the predictor variables, logistic regression achieved only moderate prediction accuracy for the five outcome states. Results obtained with discrete event simulation were comparable with logistic regression.

**Conclusions:**

Only moderate prediction accuracy could be achieved using the selected information from the Danish register RSS. Other variables need to be included in order to establish a prediction method which provides more accurate risk profiles for long-term sick-listed persons.

## Background

In the European countries the task of maintaining high labour force participation is an issue of increasing importance [1]. For the Nordic countries, in particular, a great challenge lies in the demographic development: large generations leave the labour market and small generations enter. This affects the social systems financially and will lead to a diminished labor force in the coming decades. Within the Nordic countries, the Danish model is characterized as a flexicurity model and is a unique blend of relatively high labour market participation rates, a liberal state with relatively low formal employment protection, relatively generous and accessible social benefits, and a high turnover of the work force between employments [2]. While a flexicurity model has several benefits, individuals on long-term sickness absence have little legal protection against being laid off. To counter such risks of exclusion from the labour force, intervention programs have been designed to help sick-listed employees regain their work abilities through combinations of individual and workplace interventions [3]. We aimed to develop a profiling method to allow targeted interventions for those most at risk of permanent exclusion from the job market.

Many Danish and European studies have focused on risk factors for sickness absence, on return to work, and on the consequences of sickness absence in terms of risk of future disability pension status [4-6]. Thus, much is known about risk factors for sickness absence and future disability. However, using this knowledge for the prediction of future labour market outcomes and for making a risk profile assessing the future long-term sick-listed persons to return to work, become unemployed, or qualify for a disability pension, has not been explored [7]. The development of risk profiles for individuals has gained increased attention in many countries [8]. In North America profiling is used as a tool for deciding on what, if any, program service to provide to an unemployed person [9]. A profiling tool was established in Denmark [10] to identify unemployed workers who are at risk of ending up in long-term unemployment. It has since become part of the Danish national labour market policy. An integrated part of this profiling tool is a statistical model which uses explanatory variables available from the databases of the Danish Labour Market Authorities to predict the duration of unemployment for newly unemployed workers. The purpose of the present work is to develop a prediction method for long-term sick-listed workers in Denmark, based on statistical models. The application of this method is intended to result in a profiling method with the potential to provide guidance to caseworkers in local municipalities, when making decisions regarding the allocation of resources.

The present paper will examine to what extent statistical models built on registry data can predict future labour market status for persons who are sick-listed for 4 weeks or more. Specifically, we aim to identify persons who are likely to return to work, and those at risk of unemployment, recurrent sick-listing, continuous long-term sick-listing, or early retirement. For building these models, we use the employment status after one year as the dependent variable. This variable can take one of six different values: work (W), unemployment (U), recurrent sickness absence (SA), continuous long-term sickness absence (LTS), disability pension (D), and “temporary out” (TO). As explanatory variables we use information from the Danish Register of Sickness absence compensation benefits and social transfer payments (RSS), and data from the Statistics Denmark on employment. The explanatory variables have been chosen due to their relevance for research on transitions between different labour market states [11-13] and their accessibility to caseworkers in local municipalities.

To establish a reference of comparison we apply two different prediction algorithms: a commonly used logistic regression approach and a new approach in terms of a discrete event simulation, in combination with a multi-state design and survival analysis. Both algorithms are built on a training data set and their predictive ability is evaluated in a separate validation data set; both approaches use only the information of the training data set that is available at baseline. This is in accordance with the argument that one cannot use time-varying variables in prediction, as the pathways are not known in advance [14]. The model is applied to the individuals in the test data set. The predictions of the labour market outcome one year forth, which was made from the two methods are compared in the validation set by estimates of the average Brier scores [15,16] and estimates of the Area Under the Curve (AUC) in a receiver operating characteristic (ROC) curve analysis [7].

The Study was approved by the Danish Data Protection agency. The access to the database was granted by the National Institute of the Working Environment in Denmark, Copenhagen and the Statistics Denmark.

## Methods

### Data

**Figure 1:**
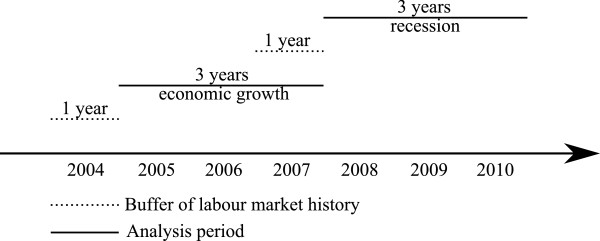
The overall study design for both the training data set and the validation data set.

The Danish Register of Sickness absence compensation benefits and social transfer payments (RSS) contains detailed longitudinal information on every sickness absence and maternity payment for all Danes in the period 2004–2010 [10]. Data from RSS were merged with individual data from Statistics Denmark on employments, socioeconomic income, and deaths in Denmark. The study sample was a drawn from a representative sample of 667,326 Danes aged 20–59 years (49.3% women) who were employed during the year 2003. This data set was divided at random into a training data set with 450,000 employees (corresponding to 67.4%) and a validation data set. With this design we are limited to comparing the prediction abilities of logistic regression and discrete event simulation when both are trained in a random sample which contains roughly 2/3 of the available data and are evaluated in the remaining 1/3 of the data. We could as well have split the data in different ways and, e.g., used 90% or 50% of the data for training. Our study design distinguishes two different macroeconomic periods (Figure 1).

The study sample consists of sick-listed individuals who experienced an initial period of long-term sickness absence (at least 4 weeks) in one of two macroeconomic periods: a period of economic growth (2005–2007) or a period of economic recession (2008–2010) [15]. In 2005–2007, 66,836 persons in the training data set and 31,567 persons in the validation data set experienced an initial long-term sickness absence period. In 2008–2010, 61,835 persons in the training data set and 29,041 persons in the validation data set experienced an initial long-term sickness absence period. The individuals were followed for one year from the date of the initial long-term sick-listing. Information about explanatory variables including employment history was collected for the 365 days preceding the date of the initial long-term sick-listing (which includes the year 2004 for individuals with the initial long-term sick-listing occurring in the year 2005). In the first year of each macroeconomic period (buffer period) no individuals were included to ensure that employment history was available for a one year retroactive period for all individuals. The two macroeconomic periods were analysed separately.

### Predictor variables

Prediction of the employment status after one year was based on the following variables, all of which were available at the time of the inclusion (the initial long-term sick-listing period): *gender*, *age* divided into four groups (20–29, 30–39, 40–49, and 50–59), *employer insurance for chronic disease* (an insurance compensating the employer from day one when the employee is sick, or used for compensation regarding absence due to periodic or continual medical examinations), *job type* grouped into seven categories (military, management, office-work, sale- service- and care taking, production and transportation, other jobs, and unregistered), *socio-economic position* grouped into four categories (self-employed, employee, without job, and unregistered), *previous sickness absence* episodes in the year preceding the long-term sickness period (grouped into “none” or “one or more”), *unemployment* episodes in the year preceding the long-term sickness period (grouped into “none”, “one”, “two or more”). These predictor variables were recorded at the first day of the initial sick-listing and not updated subsequently.

### Follow-up period

**Figure 2:**
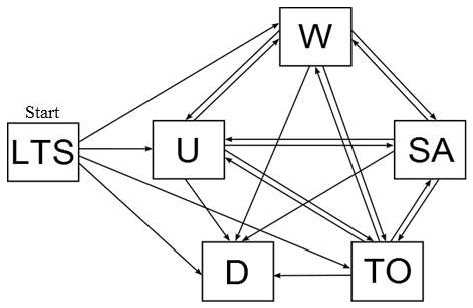
**Multi-state model, the twenty transitions between the six states: sickness absence (SA), work (W), unemployment (U), disability pension (D), temporary out (TO) and the initial long-term sick-listing state (LTS).** All individuals start in the LTS-state (marked “Start”).

Our data set includes start and end dates for all records of social payments including periods of not receiving transfer payments. From these data we derived for each individual an employment status during the year of follow-up. At any time point after the initial long-term sickness period the employment status of the study subjects was defined as one of the six states: continuous long-term sickness absence, work, unemployment, sickness absence, disability pension, or ‘temporary out’. Individuals stay in the initial long-term sickness absence state until a transition occurs. The work state consists of all time periods when no social benefit payments are registered, i.e. when the person is self-supporting or working [10]. The unemployment state was assessed as recipients of unemployment benefits either through unemployment insurance (covering 80% of the work force) or through social assistance benefits for low income households. Very few individuals in Denmark in the age range 20–59 years do not qualify for one of these benefits [10]. The sickness absence state is defined when a recurrent sick-listing is registered. During these years, Danish employees had the right to receive sickness absence benefits if they experienced sickness absence longer than 13 consecutive days. Thus, all sickness absence over 13 days is registered. Unemployed Danes also had the right to receive sickness absence benefit, but only if they were approved to receive unemployment benefit through unemployment insurance. The disability pension state is reached when the individual receives a disability benefit. Disability pension is granted when the decline in work abilities is health related and permanent. Transitions to the disability state typically come from the sickness absence state, but direct transition from the work or the unemployment state is also possible. Individuals who are granted disability pension are only available for the labour market on special terms including benefits regarding: National supplementary disability pension (early retirement pension), benefits concerning light job, flex job, or vacancy benefit for persons with a flex job. The disability state is an absorbing state from which no further transitions are possible. Persons receiving a type of social benefit that do not fit into these states are put into the ‘temporary out’ (TO) state. This includes those receiving maternity allowance and social benefits concerning education. Persons who reach the age of sixty, emigrate, or die are censured (right truncated). An overview of the states and the transitions between them is given in Figure 2.

### Logistic regression

Logistic regression was performed separately for each of the possible outcomes after one year (W, U, SA, LTS, D, TO) using the training data set. Each model included all the predictor variables in additive form.

### Discrete event simulation

The transition intensities in the multi-state model (Figure 2) were modelled by separate Cox regression models [14,17,18]. The time scale was days in the follow-up period since the initial sick-listing period. The time-varying covariates were updated at the respective entry times. Based on the training data set, we estimated the hazard ratios and baseline hazards functions of the Cox regression models for each transition. In a multi-state model framework, Cox regression results can predict short term state transition probabilities, i.e., what will happen tomorrow based on all the information available today. However, to obtain long-term predictions it is necessary to consider the Cox regression results of all possible state transitions simultaneously. For example, two persons who have the same predicted short term chance of moving to the work state will have different long term chances of being in the work state one year after the initial sick-listing, if their short term risk of moving to the disability pension state differs. Unfortunately, it is difficult to derive an explicit formula which combines the Cox regression results of all transitions in the multi-state model considered here into a prediction of the state occupation probability after one year. Instead, we developed a discrete event simulation algorithm. For an individual with a given set of baseline covariates we let Y(t) denote the state of the individual t days after baseline. The discrete event simulation algorithm is based on the following steps:

i. At time t = 0 all individuals in the validation set are in the ‘LTS’ state: Y(0) = LTS.

ii. The Cox regression results predict the short-term probabilities of the transitions from state Y(t) to Y(t + 1) where Y(t) is the state of the subject at time t and equals one of the states: LTS, work, unemployment, sickness absence, disability pension, temporary out.

iii. State Y(t + 1) is the result of a binomial experiment with the probabilities obtained in (ii). Note that also "no transition" is possible outcome of the binomial experiment. This means that the person stays in the current state.

iv. Repeat the steps (ii) and (iii) until t = 365. This yields a simulated pathway Y(0), Y(1),…, Y(365).

Repeating the steps (i) - (iv) 10,000 times yields 10,000 simulated pathways for each person. The predicted probability that a given person is in a given state, say the work state, after one year is the proportion of the 10,000 simulated pathways where Y(365) = work.

### Evaluation of prediction performance

For each individual in the validation set, we obtained predicted probabilities for the outcomes after one year based on logistic regression and based on the discrete event simulation algorithm. The performances of these predictions were evaluated using the Brier score [15] and the area under the curve estimate (AUC).

The Brier score is a residual defined for each individual as the squared difference between the observed outcome and the predicted probability. Thus, if p is the probability of being in a given state, say ‘W’ (working), after one year and the corresponding individual is working after one year, the Brier score is *BS* = (1 - *p*)^2^*, *and if the individual is not working after one year then the observed status is zero and the Brier score is *BS* = *p*^2^*.*

To compare the prediction accuracy of the two statistical models we computed the total average of the individual Brier scores in the validation data set. An average Brier score close to zero represents a good prediction model, and an upper benchmark useful for interpretation is the average Brier score of a null model which ignores the covariates and predicts the same probability for all persons in the validation data (Null model Brier score). For example if the prevalence of persons in the training data set who were in the state “W” after one year is 66.2%, then the Brier score of the null model which assigns a predicted risk of 66.2% to every individual in the validation data set is

$$\mathrm{BS}=\frac{1}{n}\left(n_{1}{\left(1-0.662\right)}^{2}+\left(n-n_{1}\right)0.662^{2}\right)=0.22$$

where *n* is the total number of persons in the validation data, and *n*_*1*_ is the number of persons in the validation data who are in the work state ‘W’ after one year. A useful prediction tool which uses the covariates to discriminate individuals at high risk from those at low risk should have a Brier score lower than 0.22.

The area (AUC) under the receiver operating characteristic (ROC) curve was regarded as a measure for discrimination. The measurement determines how well a test discriminates between those who experience a particular event and those who do not experience the event. The area is estimated under a ROC curve, which is a plot of the false positive rate (1 - specificity) against the true positive rate (sensitivity). An AUC of 0.5 indicates no discrimination above chance and an AUC of 1.0 indicates perfect discrimination. A rough guide for classifying the discriminative ability is AUC 0.9–1.0 excellent, AUC 0.8–0.9 good, AUC 0.7–0.8 fair, AUC 0.6–0.7 poor, and AUC <0.6 fail [7].

### Implementation

All analyses were done in SAS 9.2, using the LOGISTIC procedure for logistic regression and the PHREG procedure for Cox-regression [18]. The discrete event simulation/multi-state method uses the MP CONNECT (Multi-process CONNECT) feature of the SAS/CONNECT module in SAS 9.2 that allows SAS jobs to be divided into multiple independent units of work and executed in parallel.

## Results

### Descriptive tables

Table 1 describes the sample at the start of the initial long-term sickness absence period separately for the two macroeconomic periods.

**Table 1:** Descriptive table – Number of persons at the initial long-term sickness absence period in training and validation data sets, respectively

		**Economic growth**				**Recession**			
		**Training data**		**Validation data**		**Training data**		**Validation data**	
		**N**	**(%)**	**N**	**(%)**	**N**	**(%)**	**N**	**(%)**
	Total	66 836		31 567		61 835		29 041	
Gender	Women	39 988	(60%)	18 415	(58%)	36 668	(59%)	17 038	(59%)
	Men	26 848	(40%)	13 152	(42%)	25 167	(41%)	12 003	(41%)
Age group	20-29 years of age	7279	(11%)	3370	(11%)	6904	(11%)	3238	(11%)
	30-39 years of age	14 805	(22%)	7022	(22%)	15 155	(25%)	6974	(24%)
	40-49 years of age	19 644	(29%)	9330	(30%)	18 667	(30%)	8900	(31%)
	50-59 years of age	25 108	(38%)	11 845	(38%)	21 109	(34%)	9929	(34%)
Socio- economic position	Wage earner	61 810	(92%)	29 214	(93%)	57 351	(93%)	26 868	(93%)
	Self-employed	833	(1%)	385	(1%)	1447	(2%)	685	(2%)
	Without job	4191	(6%)	1967	(6%)	3015	(5%)	1481	(5%)
	Unregistered	2	(0%)	1	(0%)	22	(0%)	7	(0%)
Job-type	Military work	2038	(3%)	1014	(3%)	1467	(2%)	677	(2%)
	Management work	1103	(2%)	512	(2%)	1102	(2%)	538	(2%)
	Office-work	23 298	(35%)	10 844	(34%)	22 946	(37%)	10 720	(37%)
	Sale-, service- etc.	12 086	(18%)	5587	(18%)	10 920	(18%)	5147	(18%)
	Farming etc.	11 847	(18%)	5854	(19%)	11 086	(18%)	5155	(18%)
	Other types of work	15 137	(23%)	7125	(23%)	12 396	(20%)	5916	(20%)
	Unregistered work	1327	(2%)	631	(2%)	1918	(3%)	888	(3%)
Chronic dis.	No chronic disease	65 058	(97%)	30 793	(98%)	59 647	(96%)	27 956	(96%)
	Chronic disease	1778	(3%)	774	(2%)	2188	(4%)	1085	(4%)
Prior SA	No prior SA	42 007	(63%)	19 872	(63%)	50 671	(82%)	23 615	(81%)
	Prior SA	24 829	(37%)	11 695	(37%)	11 164	(18%)	5426	(19%)
Prior Unempl.	No prior unempl.	50 597	(76%)	23 829	(75%)	53 327	(86%)	25 008	(86%)
	1x prior unempl.	5440	(8%)	2574	(8%)	4506	(7%)	2124	(7%)
	≥2 x prior unempl.	10 799	(16%)	5164	(16%)	4002	(6%)	1909	(7%)

The sample consists of about 20% more women than men and the occurrence of the initial long-term sickness absence is higher in the economic growth period for both genders. No changes in job-type or in the proportion of people with chronic disease are seen across the time periods. A large difference between the two time periods is seen in the proportion of people with prior sickness absence and the people with prior unemployment. There is a small, expected shift in the age distribution from the first time period to the second time period and the training and the validation data sets of course show similar results.

Table 2 shows the total number of transitions in the training data set for the sample with an initial long-term sick-listing. The table is stratified by gender and macroeconomic period.

**Table 2:** The total number of transitions during each analysis period, stratified by gender

	**Women**				**Men**			
	**Economic growth**		**Recession**		**Economic growth**		**Recession**	
Transition	N	Pct.	N	Pct.	N	Pct.	N	Pct.
1: LTS → W	23 626	19.9	21 749	19.8	16 230	19.5	14 277	17.4
2: LTS → U	3002	2.5	2308	2.1	1617	1.9	1988	2.4
3: LTS → TO	7041	5.9	7775	7.1	4951	5.9	5433	6.6
4: LTS → D	210	0.2	96	0.1	153	0.2	61	0.1
5: W → U	12 113	10.2	10 847	9.9	7194	8.6	11 288	13.7
6: W → SA	17 865	15.0	15 314	13.9	14 831	17.8	10 672	13.0
7: W → TO	3468	2.9	3789	3.4	1634	2.0	1535	1.9
8: W → D	113	0.1	73	0.1	65	0.1	38	0.0
9: U → W	12 925	10.9	10 539	9.6	7515	9.0	10 229	12.4
10: U → SA	3100	2.6	3130	2.8	1542	1.8	2772	3.4
11: U → TO	708	0.6	559	0.5	239	0.3	385	0.5
12: U → D	45	0.0	31	0.0	37	0.0	24	0.0
13: SA → W	14 272	12.0	12 567	11.4	12 396	14.9	8974	10.9
14: SA → U	2979	2.5	3366	3.1	1517	1.8	2771	3.4
15: SA → TO	5533	4.7	5543	5.0	4533	5.4	3744	4.6
16: SA → D	147	0.1	125	0.1	125	0.1	93	0.1
17: TO → W	6855	5.8	7154	6.5	5060	6.1	4418	5.4
18: TO → U	1137	1.0	1258	1.1	607	0.7	1058	1.3
19: TO → SA	3791	3.2	3858	3.5	3112	3.7	2482	3.0
20: TO → D	25	0.0	18	0.0	14	0.0	7	0.0

All individuals start in the LTS-state.

The period of recession shows a slight increase in the proportion of men who experience the transition from work to unemployment, the transition from unemployment to work, or the transition from sickness absence to work. Women have a slightly decreased risk of experiencing a transition from work to sickness absence. The number of transitions in Table 2 is particularly high between the states work, unemployment, and sickness absence. Women have more transitions in to and out of the temporary out state than men – most likely due to maternity leave.

The five possible transitions towards the disability state appear less frequently than the other transitions. The most common transitions towards disability pension are those from the initial long-term sickness and from sickness absence. This is in agreement with earlier results [11].

### Results of prediction models in the training data sets

The regression parameters of the logistic regression for prediction of being in the work state are summarized in Table 3.

**Table 3:** Results of logistic regression for prediction of being in the work state one year after the initial long-term sick-listing period

		**Growth**					**Recession**				
		**OR**	**95% CI**	**p-value**	**∆AUC**	**∆Brier**	**OR**	**95% CI**	**p-value**	**∆AUC**	**∆Brier**
Gender	Male	1.25	1.20-1.30	<0.01	0.49%	0.00%	1.08	1.04-1.13	<0.01	0.00%	0.00%
	Female	1.00					1.00				
Age group	20-29 years of age	0.91	0.86-0.96	<0.01	0.81%	0.43%	0.70	0.66-0.74	<0.01	2.95%	0.43%
	30-39 years of age	1.03	0.98-1.07	0.38			0.88	0.84-0.92	0.02		
	40-49 years of age	1.25	1.20-1.30	<0.01			1.13	1.09-1.18	<0.01		
	50-59 years of age	1.00					1.00				
Socio- economic position	Self-employed	1.34	1.14-1.58	<0.01	0.49%	0.00%	0.99	0.88-1.12	<0.01	0.33%	0.43%
	Without job	0.70	0.64-0.76	<0.01			0.46	0.42-0.51	<0.01		
	Wage earner	1.00					1.00				
Job-type	Military work	0.99	0.89-1.10	<0.01	1.13%	0.85%	0.58	0.51-0.67	<0.01	0.82%	0.00%
	Management work	0.93	0.82-1.06	<0.01			0.98	0.86-1.11	<0.01		
	Office-work	0.99	0.95-1.04	<0.01			0.95	0.91-0.99	<0.01		
	Farming	0.99	0.93-1.05	<0.01			0.80	0.76-0.85	0.11		
	Other types of work	0.91	0.87-0.96	<0.01			0.73	0.69-0.77	<0.01		
	Unregistered work	-*	-	-			0.90	0.80-1.02	0.12		
	Sale- service-	1.00					1.00				
Chronic disease	Chronic disease	0.75	0.68-0.83	<0.01	0.16%	0.00%	0.72	0.66-0.79	<0.01	0.49%	0.00%
	No chronic disease	1.00					1.00				
Prior SA	Prior sick listing	0.77	0.74-0.80	<0.01	1.78%	0.43%	1.00	0.96-1.05	0.86	0.00%	0.00%
	No prior sick listing	1.00					1.00				
Prior unempl.	1 x prior unempl.	0.70	0.66-0.74	0.20	2.91%	1.28%	0.49	0.45-0.52	<0.01	3.94%	1.71%
	≥2 x prior unempl.	0.52	0.50-0.55	<0.01			0.34	0.32-0.37	<0.01		
	No prior unempl.	1.00					1.00				

*No subject returned to work.

The relative change in area under the curve (∆AUC) and change in the Brier score (∆Brier) when the covariate was removed from the analysis.

The results show that men have a better chance than women of being in the work state one year after the initial sick-listing. This result is for the economic growth follow-up period. A similar result is seen in the recession period, but the difference is much smaller. People in the age group 20–29 years have the lowest, and people in the age group 40–49 have the highest probability of being in the work state. Again results are similar across time periods, and again the differences are smaller in the recession period.

In the growth period self-employed individuals have the highest probability of being in the work state after one year, while in the second time period their probability does not differ from the wage earners. Those without job have the lowest probability of being in the work state after one year, and this difference is markedly bigger in the recession period.

People occupied by military work in the recession period have lower probability of being in the work state compared to people occupied at sale- service- and caretaking work. Comparable results are found for people occupied at farming, gardening, forestry, fishing, craftsman, and process and machinery work including transport and construction work. People occupied in other types of work have a lower probability of being in the work state after one year of follow-up in both economic periods. People with an unregistered job have a very low probability of being in the work state in particularly during the economic growth period, but since their job type is unregistered this result is difficult to interpret.

People with a chronic disease have a notably lower probability of being in the work state after one year in both time periods. People with prior unemployment have lower probability of being in the work state one year after initial sick listing, and the gradient is steeper in the recession period.

The variables were also used to predict transitions using a multi-state model (results not shown)

### Evaluation of prediction accuracy

Table 3 additionally shows the relative change in prediction accuracy of the work state, when removing one of the variables. The relative change is measured by the delta AUC and the delta Brier score. For the growth period the variables can be listed by declining relative AUC change in the following order: prior unemployment, prior sickness absence, job-type, age group, socio–economic group, gender, and chronic disease. Different results are seen in the recession period where the removal of age group had a larger effect on the AUC, though the relative change was still highest for the variable on prior unemployment. The Brier score also showed that the decline in prediction accuracy by the removal of the variable on prior unemployment is larger than the decline for any other variable.

Table 4 shows the summarized results of the predictions for all the six states – stratified by economic growth and recession.

**Table 4:** The outcome prevalence (Pre.), Area under the curve (AUC), and the Brier scores evaluated for the Null model, the Logistic prediction and the discrete event simulation

**Macro-economic period**	**State**	**Null model**			**Logistic regression**			**Discrete event simulation**		
		**Pre.**	**AUC**	**Brier score**	**Pre**	**AUC**	**Brier score**	**Pre.**	**AUC**	**Brier Score**
Growth	W	44.1%	50.0%	0.2466	43.7%	61.9%	0.2347	43.4%	62.2%	0.2344
	SA	7.8%	50.0%	0.0719	7.8%	64.3%	0.0699	6.6%	64.0%	0.0704
	LTS	4.5%	50.0%	0.0434	4.6%	58.7%	0.0432	5.6%	57.4%	0.0434
	U	5.3%	50.0%	0.0502	5.5%	72.1%	0.0477	5.0%	71.6%	0.0477
	D	0.3%	50.0%	0.0034	0.3%	73.4%	0.0034	0.7%	73.8%	0.0034
Recession	W	41.9%	50.0%	0.2435	42.5%	61.0%	0.2333	42.5%	61.5%	0.2321
	SA	9.0%	50.0%	0.0819	8.9%	61.9%	0.0806	6.7%	61.2%	0.0813
	LTS	4.0%	50.0%	0.0383	4.0%	58.6%	0.0381	4.7%	56.2%	0.0383
	U	7.5%	50.0%	0.0695	7.4%	71.0%	0.0664	7.8%	70.4%	0.0665
	D	0.1%	50.0%	0.0014	0.2%	76.6%	0.0014	0.5%	75.1%	0.0015

The estimates are shown for each of the macroeconomic periods and the prediction of being in each of the six states work (W), sickness absence (SA), long-term sick-listing (LTS), unemployment (U), and disability pension (D).

**Figure 3:**
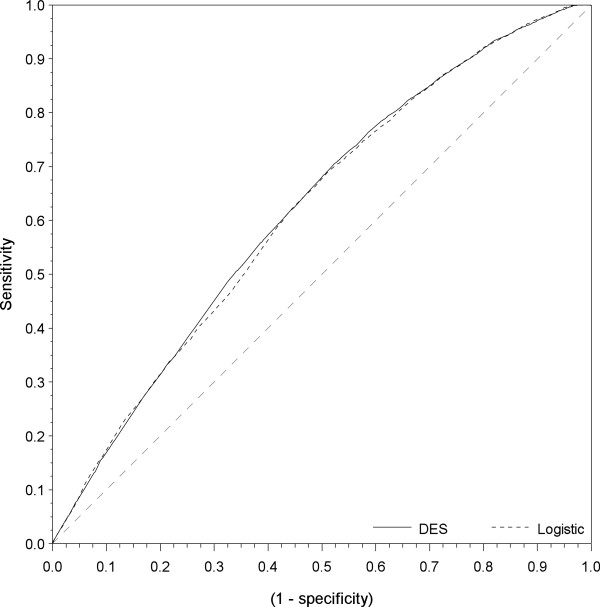
**ROC-curves for the Work-state in the economic growth period.** The figure shows the ROC-curves of the Logistic prediction method, the DES prediction approach and a reference line representing the Null-model.

When interpreting these numbers, it should be noted that the Brier score decreases with decreasing prevalence. Thus, for outcomes with low prevalence the null model Brier score is lower than for states with high occupation probabilities. For predicting work, unemployment and recurrent sickness absence both models have better prediction accuracy than the null model. For continuous long-term sick-listing and disability pension the prediction accuracies of the regression models are close to that of the null model. Thus, limited gain in prediction accuracy is shown by including the covariates. The predictions of the work status and recurrent sickness absence status are better in the economic growth period than the recession period. The predictions of the unemployment status are better in the recession period than the economic growth period. The comparison of the Brier scores shows similar prediction accuracy for the logistic regression and the discrete event simulation across all outcomes.The AUC estimates seem to fall into three groups with respect to the rough guide for classifying the discriminative ability. The predictions of the LTS state fail (<60) in both periods. The prediction of the work and recurrent sickness absence state has poor accuracy (0.6-0.7), whereas the predictions of the unemployment state and the disability state have a fair level of prediction accuracy (0.7-0.8). The AUC is generally highest in the growth period except for the prediction of the disability state. The AUC for the logistic method is slightly better than the DES method except for the prediction of the work state. In Figure 3 the ROC-curves are compared for the Logistic method and the DES method concerning the prediction of the work state in the economic growth period. Figure 3 show that the two predictions methods make highly comparable results in terms of both sensitivity and specificity.

Comparing the true prevalence with the prevalence obtained by prediction shows that: (i) both statistical methods tend to underestimate the prevalence of the work state in the economic growth period, and overestimate in the recession period; (ii) for the remaining five states the logistic approach seems to give more accurate predictions than the discrete event simulation when comparing with the true prevalence; and (iii) for the recurrent sickness absence state the discrete event simulation tends to underestimate the prevalence, and in the remaining states it tends to overestimate with the exception of the long-term sickness absence state in the economic growth period.

## Discussion

Predictions as provided by statistical models have been used in a wide range of fields to assist decision making, but prediction of future employment status for sick-listed persons has until now received limited attention. The present paper attempts to develop a method for prediction of future labour market status for long-term sick-listed Danes, based on statistical models. This paper used a large sample of Danes followed in two periods from 2004 until 2010, stratifying by economic growth and recession. Two prediction methods were compared to establish a reference of comparison, one based on logistic regression and one new approach; based on discrete event simulation, a multi-state design and survival analysis. Both methods were applied using data from one part of the sample and validated in the remaining part. Predictor variables included gender, age, socio-economic position, job type, chronic disease, prior records of sick-listing, and unemployment. For the purpose of applying a statistical model for predictions, we collected data from Danish national registries including the characteristics and employment histories from long-term sick-listed Danes. We used existing statistical models that predict the likelihood of the employment status one year later for the sick-listed individuals. In the shape of a profiling method, it was hypothesized that predictions could provide useful guidance of caseworkers at the local municipality when allocating resources.

The evaluation of the prediction accuracy showed mixed results when comparing the Brier scores and AUC with the null model. For the Brier score, the distance to the null model was relative large concerning the highly prevalent outcome of return to work, and notably low concerning the less prevalent outcomes of continuous long-term sickness absence and disability pension. The comparable values of the AUC showed poor results for the return to work outcome, failed results for the continuous long-term sickness absence outcome, but fair results for the disability outcome. This suggests that for the outcomes with a low prevalence the AUC is a better evaluation tool then the Brier score, because the distance between the Brier score and the null model has reduced sensitivity as measure of prediction accuracy as the prevalence approaches zero.

In general, we only found low to moderate gain in prediction accuracy for both statistical approaches compared to cost-free predictions (i.e., the null model which ignores the person’s characteristics and employment history). This suggests that more variables, such as diagnosis characteristics or prognosis, are needed for increasing the prediction accuracy [9]. Although the results are moderate in terms of developing a risk profiling method for long-term sick-listed Danes, it may still be possible to identify subgroups for which the gain in prediction accuracy is high.

Individuals’ employment status was followed through one year and the transitions from one status to another recorded in a so-called employment pathway. The discrete event simulation approach can be used to predict the likelihoods of the different employment pathways. Here we focused on the prediction for the status one year after the initial sick-listing period. We found similar prediction accuracies for the discrete event simulation and the logistic regression models. This indicates that for predicting the one-year outcome it does not help to study the likelihoods of different pathways towards the outcome. Hence, the simpler logistic regression approach is recommended for predicting the one-year outcome of sick listed individuals.

Evaluating the individual covariates in terms of a logistic regression shows that gender, age, socio-economic status, job type, and chronic disease all produce significant estimates but the impact on the prediction accuracy is negligible for describing people with long-term sick-listing and their probability of returning to work. The results also show the importance of including individual labour market characteristics like information on prior sick-listing and prior unemployment [12,13]. The results suggest that an increased risk of not returning to work after the initial long-term sick-listing is found in: females, younger people, people occupied in farming (recession period) and other job types, people with prior sick-listing (growth period), or prior unemployment. Further research on the topic could investigate the results of including interactions terms or investigate the effect on using stratified models. Furthermore predictions are likely to be improved by using a categorized version of variables like job-type, chronic disease, prior sickness absence, and prior unemployment, and also by using interaction effects. However, for the purpose of comparing the two different prediction methods this was not pursued here.

Three sources of bias are apparent in the presented analysis. First, invisible unemployment (unemployed persons not receiving social benefits) [19] occurs in the RSS register. This causes an overestimation of the probabilities concerning the work state. Second, when an employee gets laid off during a sickness absence period, the transition from “sick-listed employee” to “sick-listed unemployed person” is not recorded. It is likely that this has an impact on the duration of the sickness absence period and that it decreases the chance of returning to work. Third, people in the age range 50–59 years with declining health may choose to wait for early retirement pension instead of applying for disability pension. This was suggested in a forecast from the Danish Economic Council [20]. If this is the case, it would lead to overestimation of the risk of sickness absence among 50–59 year-olds.

Finally, one should be aware that the same sample is used in both periods with no new people included. This means that the sample used in the recession period is older than the one used in the growth period. Additionally the recession period includes a natural decline in the sample size, as persons who received disability pension, emigrated, or died in the growth period are not included.

These results cannot be transferred directly to other countries because of the high influence of Danish laws and regulations concerning the job market and social payments. The model containing the six states could be expanded in terms of distinguishing between the different types of unemployment compensations. This however relies strongly on both the length of the follow-up time and the size of the sample, as all the transitions must take place a minimum number of times to give reliable predictions. The model approach of the present paper follows that of Lie et al. [11], but this study’s larger sample size permitted the analysis of six states, compared to the three states analyzed by Lie et al. [11]. The larger sample size makes it possible to conduct predictions concerning events with a relatively low prevalence like disability pension. The two economic periods are only loosely defined on the basis of the OECD economic survey [21], which may cause inaccuracies in terms of deciding the precise year and date between the different periods.

The results emphasize the need for further research on the topic and on the need for including more relevant variables to the statistical models to improve prediction accuracy [9]. Long-term sick-listed individuals represent an important resource of labour, and intervention studies have shown significant effect of initiating interventions on return to work [4]. Developing a profiling method for long-term sick-listed individuals could potentially improve the success rate of an individual returning to work by providing guidance to caseworkers when allocation resources and targeting intervention programs to selected subgroups. An example of such is an automated system with a direct access to the relevant registers. Such a system could potentially make an early prioritization of cases of long-term sick-listing and place the ones with the most needs of resources in top of the caseworker to-do list.

## Conclusions

We conclude that only moderate prediction accuracy could be achieved using the selected information from the Danish register RSS. We additionally conclude that other variables need to be included in order to establish a method which provides more accurate risk profiles for long-term sick listed persons.

## Competing interests

The authors declare that they have no competing interests.

## Authors' contributions

JP was involved in the planning of the study, made the linkage and arrangement of the register data, performed the statistical analysis, and wrote the first draft and the final version of the manuscript. TAG was involved in the planning of the study, commented on the manuscript and contributed to the method and result section. JBB commented on the manuscript and contributed to the background and discussion section. KBC was involved in the planning of the study, commented on the manuscript and contributed to the introduction and the methods section.

## Pre-publication history

The pre-publication history for this paper can be accessed here:

http://www.biomedcentral.com/1471-2458/14/494/prepub
